# Dextro-Quinine

**Published:** 1879-09-20

**Authors:** George Kelly

**Affiliations:** Cleveland, Ohio


### *DEXTRO-QUININE.*

BY GEORGE KELLY, M.D., CLEVELAND, OHIO.

The high price of the old favorite sulphate of quinine, compels numbers of practitioners—myself for one—to look about and experiment with a view of discovering, if possible, some drug which shall possess the double virtue of being cheaper, and, at the same time, as efficacious as quinine sulphate. We naturally look among the alkaloids of cinchona for what we want. I tried in vain sulphate of cinchona, sulphate cinchonidia, etc., but each had some grave drawback which would prevent its coming into general use, at least it was so with me; it may be that I had always gotten hold of some inferior preparations, but this is hardly probable.

Some months ago I was induced to try the new anti-periodic, dextro-quinine, and accordingly possessed myself of an ounce and went in determined to give the stuff a fair, square trial. I will give the report of a few cases, which will serve to show my success with the alkaloid in question :

*Case* 1.—Miss Maggie McT., age, nineteen; occupation, laundress; complained of general *malaise* and a very severe headache in temporal regions every afternoon, commencing about 3 p.m., and lasting until after supper, six or seven in the evening. This condition of affairs had existed six or eight weeks; bowels regular; menses natural, etc. Put her on :

℞ Quiniæ sulph.......................................... gr. v.

Morphiæ sulph.......................................... gr. 1-16.

Ext. taraxaci.......................................... q. s.

M. fiat pil. No. 1.

Directed her to take above after each meal. At the expiration of five days she returned no better : said her headache was worse.

Discontinued above and ordered :

℞ Dextro-quiniæ.......................................... gr. v.

Morphiæ sulph.......................................... gr. 1-16.

Acid hydrochloric dil.,.......................................... q. s.

Elix glycyrrhizæ..........................................

Aqua Rosæ..........................................aa fl ℥ ss.

M. Fiat solution.

To be taken after each meal.

She returned after six days entirely cured, feels good, appetite improved, paid my bill and departed happy.

*Case* 2.—My next trial was in the case of Mrs. R., aged forty-five; married; six children; moved to Cleveland, from a very malarial district, three months ago. At about 7 p.m., May 25th, she was taken with a paroxysm of vomiting; she went to bed and immediately had a chill—to use her own words—"hard enough to shake the bed." The chill was followed by high fever and profuse sweating. All of the above symptoms subsided in due time, and next day she was apparently well, but weak. At about 6 p.m., on the third day following, she had the same chain of symptoms more marked, if possible, than before. I was then called in; found her clothing wet with perspiration; tongue coated, etc.; pronounced it a well-marked case of tertian fever, and ordered :

℞ Dextro-quiniæ.................................. gr. vij

Acid hydrochlorici dil.............................. q. s.

Syr. simplic ..............................................

Aqua dist................................................aa fl. ℥ ss.

Misce, fiat sol.

To be taken every four hours, commencing after subsidence of sweating stage.

On the third day following she had a very slight chill and fever, and some diaphoresis. Continued above ℞, and directed her to take a double dose at noon on the third day, which she did. She has had no chill or fever since. In a few days I put her on elix. bark and iron (which, by the way, is a fine preparation), half tablespoonful after each meal. She is now, June 16th, perfectly well. Mrs. R. informed me that she has had these spells for years, and resisted all treatment; she had tried "quinine, Fowler's solution, yellow puccoon bark and whisky, and everything including the celebrated Holman's Fever and Ague Pad."

*Case* 3.—Mr. Thos. M., thirty-five years of age, book-keeper, unmarried. Complained of "feeling badly" four to six weeks past; had a desire for food, but when placed before him could take but little if any of it. On May 31st had a distinct chill followed by fever, profuse sweat, headache, etc., was able to be up at his desk next morning, but, in the afternoon he had another poroxysm, fever, vomiting, sweat as before. Applied to me for relief.

℞ Dextro-quiniæ........................................ gr. xij.

Acid hydrochloric.................................. q. s.

Syr. auranti cort....................................

Aqua menthæ pip...................................aa q. s. M.

To be taken at 7 p.m., and 12 m.

He had no attack since; that is, no well-marked chill; it is true he had a slight chilliness in the afternoon after he commenced above ℞. After the fourth day I put him on smaller doses, and he reports himself well.

The above cases, the record of which may not be so minute as desirable, will show with what degree of success I have used the new remedy.

Now a word in regard to the preparing of dextro-quinine for administration :

I like it best when combined with muriatic acid, because it is, in my opinion, the best solvent for the drug and renders it very acceptable to the stomach. The addition of elix. glycyrrhiza makes it a very nice and not unpleasant mixture. After the use of dextro-quinine I failed to find any of the disagreeable sequellæ characteristic of sulphate of quinine. As I believe dextro-quinine to be the equal of sulphate of quinine, as an anti-periodic, is less than half as costly, makes a much more pleasant mixture and produces no tinnitus aurium, as is the case with sulphate of quinine, I have determined to adopt it in my practice, and believe it will not disappoint me in what I expect of it.

It may be well to add that the dextro-quinine I use is from Keasbey & Mattison, Philadelphia, and I think that they merit the thanks of the profession for placing within our reach an article which is inferior to sulphate of quinine in no particular, and superior to it in many points. —*Lancet and Clinic.*

